# What do we think we are doing? How might a clinical information network be promoting implementation of recommended paediatric care practices in Kenyan hospitals?

**DOI:** 10.1186/s12961-017-0172-1

**Published:** 2017-02-02

**Authors:** Mike English, Philip Ayieko, Rachel Nyamai, Fred Were, David Githanga, Grace Irimu

**Affiliations:** 1 KEMRI-Wellcome Trust Research Programme, P.O. Box 43640, Nairobi, 00100 Kenya; 2 Nuffield Department of Medicine, University of Oxford, Oxford, United Kingdom; 3 Maternal, Newborn, Child and Adolescent Health Unit, Ministry of Health, Nairobi, Kenya; 4 Department of Paediatrics and Child Health, University of Nairobi, Nairobi, Kenya; 5 Kenya Paediatric Association, Nairobi, Kenya

## Abstract

**Background:**

The creation of a clinical network was proposed as a means to promote implementation of a set of recommended clinical practices targeting inpatient paediatric care in Kenya. The rationale for selecting a network as a strategy has been previously described. Here, we aim to describe network activities actually conducted over its first 2.5 years, deconstruct its implementation into specific components and provide our ‘insider’ interpretation of how the network is functioning as an intervention.

**Methods:**

We articulate key activities that together have constituted network processes over 2.5 years and then utilise a recently published typology of implementation components to give greater granularity to this description from the perspective of those delivering the intervention. Using the Behaviour Change Wheel we then suggest how the network may operate to achieve change and offer examples of change before making an effort to synthesise our understanding in the form of a realist context–mechanism–outcome configuration.

**Results:**

We suggest our network is likely to comprise 22 from a total of 73 identifiable intervention components, of which 12 and 10 we consider major and minor components, respectively. At the policy level, we employed clinical guidelines, marketing and communication strategies with intervention characteristics operating through incentivisation, persuasion, education, enablement, modelling and environmental restructuring. These might influence behaviours by enhancing psychological capability, creating social opportunity and increasing motivation largely through a reflective pathway.

**Conclusions:**

We previously proposed a clinical network as a solution to challenges implementing recommended practices in Kenyan hospitals based on our understanding of theory and context. Here, we report how we have enacted what was proposed and use a recent typology to deconstruct the intervention into its elements and articulate how we think the network may produce change. We offer a more generalised statement of our theory of change in a context–mechanism–outcome configuration. We hope this will complement a planned independent evaluation of ‘how things work’, will help others interpret results of change reported more formally in the future and encourage others to consider further examination of networks as means to scale up improvement practices in health in lower income countries.

## Background

Evaluations of district hospitals in low- and middle-income countries have shown that the care provided in many clinical areas is of inadequate quality [1–3]. This is often linked to poor infrastructure and limited material and human resources. However, it is also observed that the technical process of care is often inconsistent with evidence-based recommendations that are designed with resource constraints in mind [4]. There are clearly many potential explanations for failing to provide recommended forms of care even when resources are available; these range from inadequate knowledge to poor motivation to embedded practice norms that conflict with recommendations [5–8]. Overcoming such an array of challenges is implicit to calls for improved translation of knowledge into practice or implementation of essential interventions at scale. Many tests of implementation tend to focus on a specific intervention or tackle a single illness or a carefully delineated small set of challenges. Building on earlier work [9, 10], we designed [11], and have now implemented, a possible broader strategy aimed at improving adoption of multiple evidence-based guidelines for paediatric hospital care in Kenya. Here, we summarise our original intentions and outline the activities that have comprised this strategy for a period of over 2 years. We then re-interpret our intervention strategy by reflecting on how its components align with a recently published typology [12] before exploring how these components may leverage change using a second comprehensive framework, the Behaviour Change Wheel [13]. Finally, we consider how we might reframe our strategy in realist terms as a mid-range proposition or theory [14]. As implementers of the strategy ourselves, we aim to make our assumptions more transparent so that these can be contrasted with an independent evaluation of the strategy through the eyes of its targets (work in progress). We hope this presentation of our activities and thinking will also facilitate interpretation of reports more fully describing changes in care within the established Clinical Information Network (CIN), as well as contributing to wider thinking on the nature of clinical networks and their potential role in improvement in low-income countries.

### The rationale for a ‘network strategy’ to improve care

A full explanation of how the proposed strategy for intervention might tackle identified challenges is provided elsewhere [11]. The broad strategy was based on considerable prior experience of the context, the clinical focus of care and our understanding of theory that resonated with challenges diagnosed before initiating the current intervention approach. In brief, constraints operating to undermine the provision of care in accordance with evidence-based clinical practice guidelines were felt to include failures of expert professionals (paediatricians) to take a more holistic responsibility for paediatric service delivery beyond their personal technical contribution to individuals’ care. With typically only one or two of these personnel in a hospital and with a form of training that emphasizes expert knowledge, this failure resulted, in part, from their being inadequately prepared and poorly supported to act as leaders of service units (sometimes referred to as hybrid clinical managers [15]). This was compounded by their often being professionally and geographically isolated. Further exacerbating these system deficiencies was an almost complete lack of useful information for routinely monitoring or understanding care processes or outcomes within hospitals [16]. We therefore proposed bringing together efforts to disseminate national policies, the national professional association and implementers (a research group) to develop a collaborative network to tackle these constraints. As part of this, we aimed to provide paediatricians with simple skills to act as effective hybrid managers when engaging with senior managers and frontline staff to improve services. In summary, our proposed network would act as a system oriented intervention targeting the pivotal role of leaders of service units [15], while also addressing the wider professional context (or norms) likely to be an important influence on practice [17]. The network was also to generate improved information on the process of care being provided and share this through episodic feedback. This feedback is linked to discussions with senior clinicians and is intended to promote adherence to national clinical guidelines, thus attempting to make use of likely positive benefits of feedback and outreach [18, 19]. Having articulated what we intended, the actual evolution of network activities is now described.

## Methods

We (ME, PA and GI) first utilised research records, prospectively maintained administrative logs capturing the dates and names of activities, and field diaries kept by key implementers to construct a temporal sequence of key activities that together have constituted network processes over 2.5 years.

Using this characterization of the CIN’s activities we specifically wished to describe the implementation effort as understood by the implementing team itself drawing on literature and our experience of being part of the intervention. In this sense, our efforts are analogous to describing from an ‘insider’ perspective our emergent theory of change based on the reality of implementation. Our specific intention to present an insider perspective was based on a desire to answer the question ‘What do we think we are doing?’ In separate work (ongoing), investigators that are not part of the implementing team are examining the perceptions of intervention recipients. Our rationale for keeping these investigations separate is to explore coherence or divergence in the pictures that emerge that may shed light on the relative importance of specific intervention strategies (for example, whether our categorisation of major and minor is meaningful) and provide some insight into the fidelity of our characterisation of the intervention approach. Therefore, the process of elucidating our understanding of the intervention included only the authors and three further members of the implementation team as participants in an iterative process of review and discussion. During this process, academic colleagues were consulted to help inform our understanding of key literature, to clarify ideas and to help build consensus where necessary. The implementing team were aware of the emerging quantitative data spanning a number of indicators used to measure effects of the CIN that were the subjects of feedback to hospitals. However, this report is not aimed at trying to present or explain these quantitative findings formally. Instead, it is aimed at better defining the intervention so that more formal mixed methods analyses examining intervention effects are possible, analyses that will include insights gained from intervention recipients.

The approach proceeded in stages. Our first step was to critically analyse the CIN activities to distil their central active ingredients using a recently published typology of implementation components as a reference to give greater granularity to this description [12]. To achieve this, the reference material was circulated to the group (n = 9) 2 weeks before one author (ME) circulated an initial proposed listing of CIN intervention strategies. Over a subsequent period of 8 weeks, group members met and made comments on successive drafts of a final listing until consensus was reached. As part of this process we classified intervention strategies employed as either major or minor. We believed major strategies to be a central component of the intervention specifically aligned with CIN activities. Minor strategies were those that the group felt were employed but only partially executed or that were implemented as a by-product of the overarching network approach without being specifically planned. As part of this process, we identified strategies that were clearly not relevant to the CIN as an intervention. In some cases, strategies were discussed as potential minor intervention components but were not felt to meet the criteria of being actively and sufficiently employed within the network. A final category of strategies were those that were historically related to the CIN intervention but not a current active component. In response to peer review comments, this stage of the process was revisited and refined in two ways in a new round of consultation. First, specific reference was made to supplementary material provided by Powell et al. (Appendix 6 linked to [12]) that offers more detail on definitions of the 73 intervention strategies. Second, we adopted the conceptual groupings of all 73 implementation strategies defined by Waltz et al. [20] to organise our final list of the strategies we believe are encompassed in the CIN intervention.

In a next step, we used the Behaviour Change Wheel [13] to propose how the network activities and linked intervention strategies we had articulated may operate to achieve change in frontline health workers behaviour. This exercise was again based on review of the primary reference, reflection and discussion in meetings until consensus was reached on the policy categories, intervention functions and sources of behaviour (as defined by Michie et al. [13]) that we believe are influencing participants in the CIN.

To illustrate from our insider perspective how intervention strategies and behavioural influences may produce effects within the CIN we present results from two specific indicators of practices the CIN was aiming to influence that have been tracked since the inception of the network as simple time-series graphical plots. It is important to note that these are just two measures of many indicators being measured across the illnesses targeted for improved care within the network and are used for illustration only. A fuller account of how quantitative measures of care changed over time will be provided elsewhere. In a final step in the process, presented in the discussion, we (ME and GI) attempted to synthesise in more summary form our understanding of the way our network may be achieving change by formulating a realist context–mechanism–outcome configuration [14].

## Results

### Activities conducted as part of the network intervention

In Table 1, we summarise the activities that have been conducted across the network between its inception, in September 2013, to April 2016. At the centre of the network was a senior clinician coordinator who, in addition to the events outlined in Table 1, made three further trips to each hospital within the first 15 months to provide face-to-face feedback and discuss hospital feedback reports. This process also acted as a form of coaching to local paediatricians expected to deliver the feedback to local teams themselves as the CIN continued. A data coordinator who worked with the one data entry clerk at each hospital also visited each site every 3 months to reinforce good data collection practices. Every second visit (6 monthly) this data coordinator conducted a verification of the data being entered by independently entering data from a random set of records and contrasting entries with those of the local clerk providing feedback and reinforcing training as required. (A full description of the data collection process is provided elsewhere [21, 22]). The clinical coordinator and the data coordinator also maintained phone and email contact (approximately every 2 weeks) with the paediatricians and data clerks, respectively. Between January 2014 and March 2016 quality of care reports spanning a summary of admission diagnoses, outcomes and reporting on the quality of documentation in the medical record have been sent to hospitals 10 times (approximately every 3 months). These reports also provide information on whether the care provided (documented) adheres to key aspects of national guidelines for malaria, pneumonia, diarrhoea and dehydration, severe malnutrition, and meningitis.

**Table 1:** A summary of major activities conducted as part of developing the Clinical Information Network (CIN) between September 2013 and April 2016

Timing	Activity
Sept 2013	Introductory meeting with paediatricians from potential CIN hospitals (1 day)
	Training of data clerks with hospital health record information officers (3 days)
	Data collection initiated in eight sites with introductory (1 day) training provided for clinical teams in their own hospital
Feb 2014	Training of data clerks with hospital health record information officers (3 days)
	Data collection initiated in six new sites with introductory (1 day) training provided in their own hospital for clinical teams
May 2014	First CIN meeting (2.5 days); from each hospital: paediatrician, nurse lead for paediatrics, health records information officer. Areas covered: understanding the CIN quality of care reports; basic introduction to team leadership
June 2014	Clinical coordinators visit all sites to provide half day meetings presenting hospital-specific feedback and discussion of these feedback reports
July 2014	Database familiarisation and analysis workshop for Health Records Information Officers from CIN hospitals (2 days)
Oct 2014	Second CIN meeting (1.5 days); paediatricians only. Areas covered: understanding the CIN quality of care reports; comparing performance across hospitals; priority setting for improvement in each hospital
	Explanation of evidence supporting proposed new pneumonia guidelines (1 day); five CIN paediatricians joined national guideline review panel for pneumonia
Nov 2014	Refresher training provided for CIN hospital data clerks (1 day)
Jan 2015	Database familiarisation and analysis workshop for Health Records Information Officers form CIN hospitals (2 days)
June 2015	Third CIN meeting (1.5 days); paediatricians only. Areas covered: understanding the CIN quality of care reports; comparing performance across hospitals; the principles of feedback and how to make it effective
	CIN paediatricians also each joined one or more national guideline panel reviewing evidence and making recommendations on common newborn care national guidelines (1–2 days)
Oct 2015	Fourth CIN meeting (2.5 days); paediatricians, nurse lead for paediatrics, health records information officers. Areas covered: CIN quality of care reports; comparative performance across hospitals; specific additional analysis on blood transfusion practices, monitoring of vital signs and treatment of shock Discussions on standards of care to improve monitoring of vital signs, checking for blood glucose in serious illness, checking HIV status on all admissions and improving recording of discharge diagnoses
Feb 2016	Pneumonia clinical guideline change training (1 day); delivered by one of CIN team members at each hospital to clinical and nursing teams

### Interpreting our network intervention using a recent typology of implementation intervention strategies

We briefly outlined a rationale for the network we intended to deliver as an intervention strategy in the introduction. In the first part of these results, we presented a brief description of the activities conducted as part of the network intervention. Here, we examine the intervention efforts to identify the specific implementation strategies encompassed by these activities [12]. To do this, we tabulate these strategies, organised within conceptually related groups as defined by Waltz et al. [20], and indicate our rationale for considering these to be active ingredients of the network intervention being delivered (Table 2). This process suggests that we are employing 22/73 defined implementation strategies [12]. Amongst these we would consider 12 to be major active components and 10 to be minor.

**Table 2:** A summary of major and minor intervention components encompassed within the overall network intervention strategy drawing on a recent typology [12] of specific strategies and organised in line with conceptual domains linked to this typology [20]. A brief description of the form that these intervention components took as the network was implemented is also provided

Intervention components	Operational form within the network intervention strategy
1) Alter incentive structure^a^	Recognition by the coordinating team and peers of good service provision and achievements in improving care by local teams led by the paediatrician while conversely making it a matter of concern if there is poor care in relation to shared professional goals/standards (assessed using agreed indicators) while avoiding embarrassment/humiliation
Domain: Change Infrastructure	
2) Change record systems and 3) Mandate change^b^	Work with partners was conducted to implement standardised components of medical records including admission clinical forms (checklists); network meetings provided a forum to discuss and promote consensus amongst peers in the presence of a small number of senior members of the paediatric community on the need to promote nationally recommended practices in the form of agreed national guidelines
Domain: Use Evaluative and Iterative Strategies	
4) Audit and provide feedback, 5) Develop and implement tools for quality monitoring, and 6) Develop and organise quality monitoring system	Building a mechanism for capturing trustworthy data that enables measurement of practice against relevant and agreed indicators supported by the introduction of a standardised admission record form that enables data capture and subsequent analysis based on indicators of adherence to guidelines and regular reporting on these indicators (feedback) to hospitals in the form of performance reports sent to team leaders at the end of every 2–3 months
Domain: Provide Interactive Assistance	
7) Facilitation (external)	At the network centre is a clinical coordinator who coordinates network meetings and transmits the feedback by email and then discusses it by telephone, providing advice as required while also promoting peer-to-peer support; the clinical coordinator visited each hospital 2 to 3 times in the first 12 months of network activity
Domain: Develop Stakeholder Interrelationships	
8) Build a coalition, 9) Promote network weaving, 10) Develop academic partnerships, 11) Conduct local consensus discussions, 12) Inform local opinion leaders^b^, 13) Involve executive boards^b^, 14) Recruit, designate, and train for leadership^b^, and 15) Capture and share local knowledge	Deliberate effort to create a network (“*a grouping that aims to improve clinical care and service delivery using a collegial approach to identify and implement a range of* [improvement] *strategies*” [38]) that spans government, a professional association, senior peers and hospital team leaders; this is linked through meetings, clinical network coordinator to paediatrician contacts and an informal peer-to-peer messaging group offering a forum to share experiences and learning with a focus on promoting local leadership at the middle (clinical) level of management and change efforts to promote guideline adherence and care improvements. The network itself is built on existing relationships across government, the professional association, a university and a research institution, and with paediatricians who often had training in the university or who share professional ties. These groups have also helped create national guidelines for all hospitals (outside the network too). At the centre of the network are a research institute and a representative of the university who provide the data management and analytic support linked to regular feedback and outreach (as above) to county hospital paediatric teams. As typically there is only one paediatrician and one nurse and information officer leader, the network team had no active role in recruiting or designating team leaders Early stakeholder meetings with government and hospitals’ management teams, and especially paediatricians, were conducted on a background of longer term sensitization to the problem of quality care and the value of evidence-based clinical guidelines created with the Kenyan paediatric community. In the 4–6 monthly face-to-face network meetings with paediatricians, their responsibility for improving practices was discussed with simple training provided (see below). Through a WhatsApp group, coordinator encouraged sharing of stories on how hospitals have promoted change
Domain: Train and Educate Stakeholders	
16) Create a learning collaborative, 17) Providing ongoing consultation, 18) Conduct educational meetings^b^, 19) Make training dynamic^b^, 20) Distribute educational materials^b^	The network was initiated with a meeting of a paediatrician, a senior nurse and the health records information officer from each participating hospital and the research institute and university partners. At this meeting and at subsequent hospital-specific introductory visits the network was explained and its purpose to promote better generation and use of health information to support better care. Collaboration is supported from the network centre by a clinical coordinator who coordinates network meetings and offers the feedback, discusses it and provides advice as required while also promoting peer-to-peer support. During the 4–6 monthly meetings predominantly with paediatricians specific short sessions (< half a day) were provided that explained leadership of teams, how to give group feedback, on understanding complex systems and on the principles of quality indicators and their use. The training typically used discussion, reflection and individuals’ experiences as well as presentations. Relationships and the educational approach were complemented by visits to hospitals by the clinical coordinator in the first year to explain and discuss the hospital-specific indicators provided in an overall report National guidelines for care were distributed to network and non-network hospitals as part of a national distribution and some network paediatricians took part in updating these guidelines in 2015
Domain: Support Clinicians	
21) Revise professional roles^b^, 22) Facilitate relay of clinical data to providers^b^	There has been no formal effort to revise or codify the professional role of the paediatrician or influence accreditation processes (Kenyan paediatricians do not undergo regular reaccreditation once registered with the medical board) but the network aims to foster a shift of role norms for paediatricians through social influences. This consultant group is largely trained to be expert diagnosticians and managers of therapeutic care at individual level and developing an expanded role concerned with overall patient service delivery and quality of care with a responsibility for clinical team performance is implicit in the network approach. There remains no facility for immediate or rapid relay of data to clinicians or their team leaders in the form of decision support or an interface providing on-demand reports but 2 to 3 monthly reports are regularly delivered

^a^Altering incentives is described in a purely financial sense in the original typology within the domain ‘Utilise financial incentives’. As enacted in our network the approach to altering incentives we used did not align well with this or other domains

^b^Minor components

Amongst the implementation strategies as defined by Powell et al. [12] and conceptually grouped by Waltz et al. [20], we did not feel the CIN actively employed strategies in the domains of ‘Adapt and Tailor to the Context’, ‘Engage Consumers’ or ‘Utilize Financial Strategies’. In the case of Tailor Strategies (defined as tailoring to ‘address barriers and leverage facilitators that were identified through earlier data collection’) this decision was debated. However, our rationale was that, although the CIN was developed based on considerable prior work spanning over a decade [1, 5, 10, 23] that informed initial theorising on intervention design [11], we did not collect new data to support this theorising before initiating the intervention.

Within CIN, we felt that ‘Alter incentive structures’ was a major component implementation strategy. In the typology developed by Powell et al. [12] this is defined as “*Work to incentivize the adoption and implementation of the clinical innovation*”. However, its further description makes it clear that it refers to purely financial incentives and, in the classification by Waltz et al. [20], this strategy is conceptually grouped in the domain ‘Utilise Financial Strategies’. We believe this points to a potential gap in the existing typology that seems to exclude socially constructed incentives such as recognition and promoting professional values that may have important effects on behaviour [24]. We therefore retain ‘Alter incentive structures’ as a major component but do not classify it within the currently proposed domain structure (Table 2).

Given the broad nature of our network approach judgements had to be made about whether or not we were actively delivering specific components. This was of particular relevance to our identification of minor intervention components and those not felt to meet the criteria of being actively and sufficiently employed. For example, we felt the network had little role in ‘Change physical structure and equipment’ as any organisational changes were left to local hospitals and no direct financial support was provided. This decision was made although we did supply a single desktop computer for data entry and advocate with national health sector partners for provision of mid-upper arm circumference (MUAC) tapes to screen for malnutrition. However, the single desktop was used solely for data collection within the records department and we considered this to be part of the strategy ‘Develop and implement tools for quality monitoring’. We did not consider advocating for delivery of a job aide for a very specific task as a sufficient criterion of ‘Change physical structure and equipment’ in hospital paediatric units, although for this very particular task it may have supported change (as described below). Similarly, we did not feel our approach helped ‘Identify and prepare champions’ as the leaders we worked with were selected by virtue of their existing, formal position and we were engaging them in a process where they might reconsider the scope and responsibilities associated with their established role. Instead, we felt our efforts to support clinical leaders was best captured within a minor intervention component as ‘Recruit, designate, and train for leadership’. This component was considered minor as we had no role in recruitment or designation, we worked with clinical leaders already found in participating hospitals, but we did offer some training in a role as clinical leaders (Table 2). At a higher level, although the CIN intervention is being undertaken in partnership with the national paediatric association, it did not have the authority to ‘Change accreditation or membership requirements’. Instead, our efforts to change professionals’ behaviour we feel is being mediated through two minor intervention strategies, the creation of a learning collaborative and use of social influences to revise professional roles (Table 2).

### Changing behaviours in hospitals within the network

Above we have articulated what implementation components are included as part of our CIN intervention. However, such a process of deconstruction may not help us understand how these components work together to effect change. Change that is essentially the consequence of individual and group behaviours. Here, we use the Behaviour Change Wheel framework to explore how these behaviours may be influenced [13]. In line with this framework, we consider policy level levers and forms of intervention and their effect on capability, opportunity and motivation. At the policy level the network promoted and helped distribute clinical guidelines with hospital paediatricians themselves engaged as members in at least one national guideline development panel in 2015. This exposed network partners to the process of evidence-based guideline development [25] and may have built trust in their value. The network also acted as a means of communicating and marketing these guidelines and feedback within the network focused on hospitals’ compliance with them, feedback that was shared across the peer group involved in the network.

The Behaviour Change Wheel describes a number of forms of intervention. We feel the network employs Incentivisation, Persuasion, Education, Enablement, Modelling and Environmental Restructuring. Perhaps the main form of incentivisation (as in Table 2) is the effort to use recognition of successful change and improvement efforts and link this to promoting professional pride in these successes. Persuasion is employed across the collaborative approach spanning engagement in guideline development, links to authoritative organisations, including the research institute, the university and the national paediatric association, and the use of processes that encourage development of shared goals and peer-to-peer benchmarking and interaction. The engagement of multiple institutions and peers across the network provided opportunities for education, particularly focused on developing understanding of new roles and areas requiring improvement (as distinct from formal skill oriented training). Modelling by providing examples for people to imitate and enablement such as that provided by the continued support of the clinical coordinator were also employed. No restructuring of the physical environment in participating hospitals was conducted by the network team, but we did change the medical record systems and furnish information where it did not exist. This information on adherence to guidelines is aimed at creating a professional and social expectation to improve. Linked to a sharing of this information across groups and peers we were also aiming to create new norms and to revise socially constructed professional roles.

Ultimately, these strategies are anticipated to promote motivation, enhance psychological capability and provide the social opportunity that will create change. The effect on motivation of the intervention described is mainly perhaps through the reflective pathway with participants engaged in developing plans and evaluating progress against goals that are shared with experts and peers (in common with more specific quality improvement strategies [26]). However, we believe automatic motivation may be triggered by a link to an innate desire to provide good care encompassed in the idea of vocation amongst health workers [27]. The engagement in creating guidelines, understanding indicators, and involvement in reflection on and construction of approaches to change practice might improve the psychological capability of both individuals and the network as a group. Meanwhile, an overarching theme of the network is to change the social milieu in which the clinical leaders in hospitals operate. A broad professional focus on adoption of guidelines and improvements in care that is endorsed by recognised institutions and the professional association, as well as an effort to create ownership of this agenda, are at the heart of the network strategy.

### Examples of change within the network

An early focus of the network was to improve the availability and quality of accessible data to characterise care quality for inpatient children. The challenges to be overcome and the approach taken have been described elsewhere [21]. In brief, medical records themselves were often poorly kept and medical documentation was limited in scope and quality. Routine health information systems focus only on aggregate workload and morbidity and mortality statistics and are not designed to provide any information on quality of care. Furthermore, weaknesses in the information system [16] and its predominant role as a means to send data to a national repository with little use of data locally have created a system of care that is rarely informed by data analysis. By fostering introduction of (but not providing) structured forms to standardise the content of inpatient records [28], collating and analysing the data they contain, and providing feedback on data quality, clinical documentation has considerably improved [22].

**Fig. 1:**
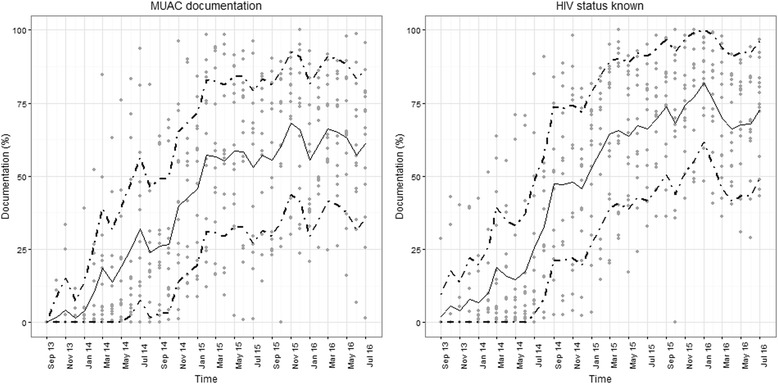
Scatter plots showing each hospitals’ performance (*grey circular markers*) each month from September 2013 to July 2016 for documentation of HIV status (panel **a**) and documentation of the result of screening using mid-upper-arm circumference (MUAC, panel **b**). The solid central trend line represents the median value of the 14 hospital specific observations and the *upper* and *lower dotted trend lines* represent the *upper* and *lower* interquartile range of the 14 hospital specific observations, respectively

Early efforts to promote adoption of guideline recommendations included focusing attention on ensuring all admitted children had their HIV status ascertained. Knowing the HIV status is important in determining treatment of acute illnesses (e.g. pneumonia) and has become increasingly important as now all children who are positive, irrespective of immune status, should be started immediately on antiretroviral medication. Documenting HIV status was discussed at network meetings and feedback on hospitals’ performance was provided in regular reports (Tables 1 and 2) with paediatricians encouraged to overcome local barriers to making testing more available and to promote documentation of status by their clinical team members to avoid missed or multiple testing. This has seen a steady rise in clearly documented HIV status over time (Fig. 1a). In 2013, new national guidelines recommended that sick children be screened for malnutrition using measurement of MUAC. It was clear from the data, discussions at network meetings and with the clinical coordinator that this new policy was not being enacted. The initial problem being one of supply of simple MUAC tapes designed for the purpose. Network partners began to advocate with the national Ministry of Health and its partners to make these tapes available. Once this happened, feedback on use of this screening tool, linked to ongoing discussion at network meetings and through the coordinator, was used to promote its uptake (Fig. 1b). These examples we hope help illustrate some of our decisions on what we consider active implementation strategies encompassing ‘Change record systems’, ‘Develop and organize quality monitoring systems’, ‘Develop and implement tools for quality monitoring’, ‘Audit and provide feedback’, ‘Create a learning collaborative’, ‘Build a coalition’, ‘Revise professional roles’, and ‘Mandate change’ (Table 2).

## Discussion

We have used work on a recently published typology of intervention strategies [12, 20] to characterise a broadly based network intervention approach aimed at improving hospital care for children in Kenya. In doing this, we have illustrated some of the decisions we made, decisions some might contest. Of nine conceptual domains that usefully categorise 73 intervention strategies we feel the CIN approach employs components from across six domains. We were unable to utilise financial strategies and did not attempt to engage consumers in this low-income setting. We felt efforts to tailor the intervention approach were not an active part of the CIN intervention but that this was very much informed by earlier work. In general, the CIN intervention seems to draw most heavily on the conceptual domains ‘Use evaluative and iterative strategies’ (three major components), ‘Develop stakeholder interrelationships’ (four major and four minor components), and ‘Train and educate stakeholders’ (two major and three minor components) from across the 12 major and 10 minor components we identified. In conducting this work a potential limitation of the typology used appeared. It seems that the intervention strategy encompassing altered incentives is defined to specifically include financial incentives (Alter incentive/allowance structures) and is conceptually mapped to the ‘Utilise financial strategies’ domain [12, 20]. We slightly amended the name of this intervention strategy to ‘Alter incentive structure’ to encompass non-financial incentives we and others believe may be important [24]. In the specific case of the CIN we aim to leverage the recognition of senior colleagues and peers linked to reaffirming professional values as incentives.

To help create a bridge between this effort to identify the network intervention’s constituent parts and proposed mechanisms of action we have used the Behaviour Change Wheel framework [13]. This provides one with means of articulating how interventions may influence individuals’ capability, opportunity and motivation to change their behaviour, ultimately the purpose of the network. Our rationale for explaining what we consider to be what we are doing is to enable others to critique our assumptions. As the authors are a core part of the network and implementation team, such external views may be illuminating. We also provide a link between our original efforts to explicate an intervention design to meet the challenges felt to be present in the Kenyan context [11] and the practical consequences of implementing this design thinking. In essence, we are articulating an emerging theory of change from an insider perspective. As we move beyond the examples of change illustrated here to report more formally what changes the network may have enabled across multiple clinical conditions we also hope this articulation of the network processes will help people understand both what was done and consider whether any effects reported in the future are plausibly related to the approach. Additionally, we anticipate that an independent, qualitative evaluation of the network will examine its effects from the perspective of key hospital participants. We wished to state our expectations of how the network may be operating in advance of this evaluation to allow for a more fruitful comparison.

This report is not aimed at presenting or claiming that the network is an effective mechanism for change, although we do present examples of the types of change we are interested in to illustrate the application of intervention strategies. There are clearly many difficulties in making claims of cause and effect convincing; we do intend to present a more specific evaluation of network effects in due course drawing more fully on mixed methods approaches to evaluation. Such mixed methods approaches may help overcome, to some extent, the considerable challenges in meeting current (and appropriate) standards for evidence of cause and effect when examining complex interventions. In the case of a network as intervention it is of course hard to provide an appropriate counterfactual in a temporally parallel experimental design. In essence, we only have one unit of intervention and analysis – the entire network – although this spans multiple individual hospitals. It is not entirely clear that a set of hospitals outside a network are a grouping that provide an appropriate comparison. Clearly, the intervention itself is highly complex and this provides further challenges in teasing out cause and effect relationships [29, 30]. Alternative evaluation designs might include a stepped-wedge strategy to implement the network. However, as one key feature of a network is establishing partnerships, sharing learning and developing a peer-to-peer group, any experimental advantage of this design would likely be undermined by its potentially detrimental effect on the intervention process and thus effect. Time series approaches offer perhaps the best prospect of exploring change and its association with network participation [31], although as networks such as ours tackle a number of diverse areas measured by a multitude of indicators, conducting such studies is also not without challenge. However, rather than evaluating the effect of a network compared with its absence opportunities exist for examining change within the network and spread of innovation over time. Thus, we hope to test the value of alternative feedback strategies, recognised as a current research need [32], by randomising hospitals already within the network to different feedback interventions.

**Fig. 2:**
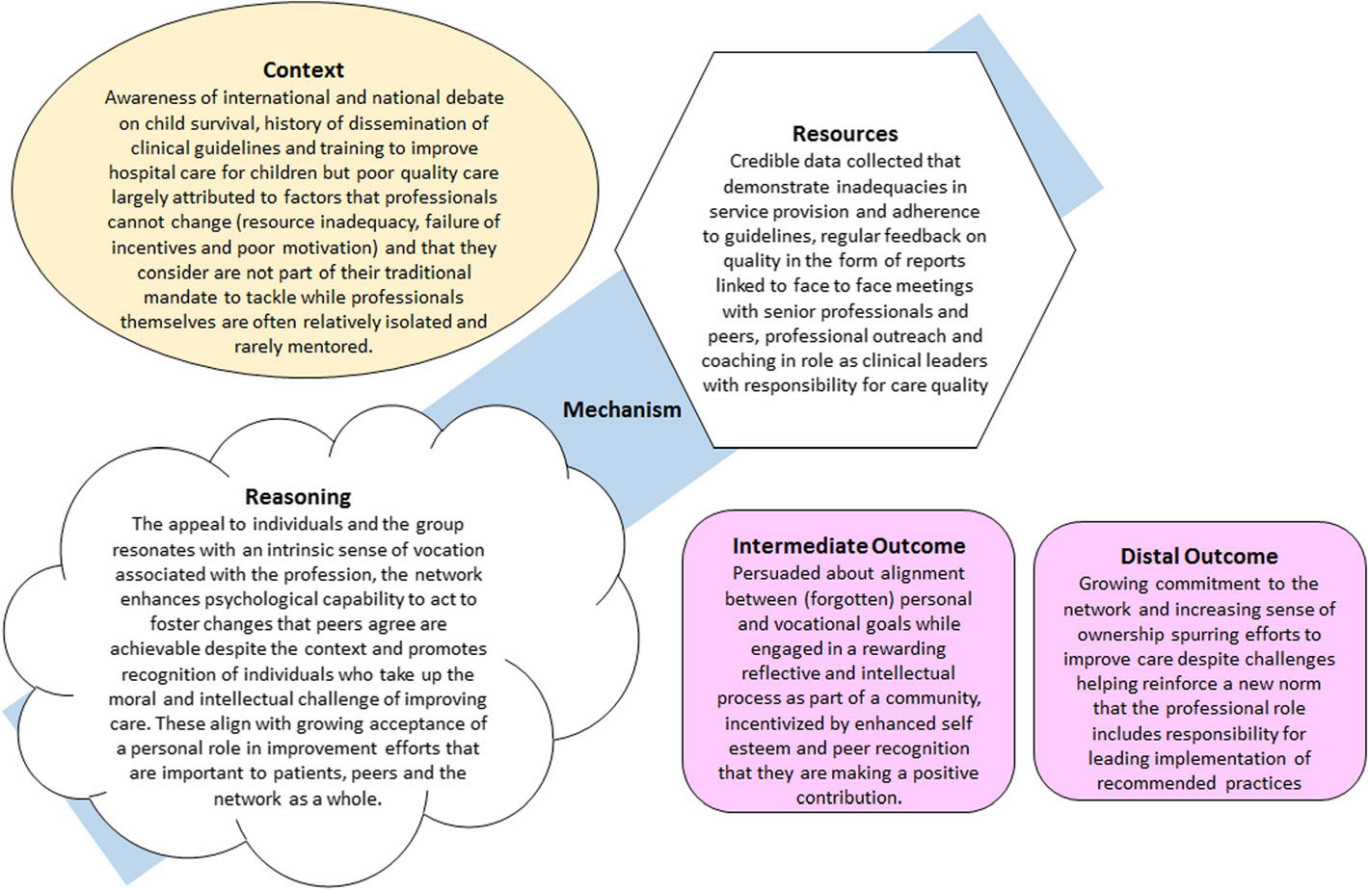
Context–mechanism–outcome formulation of the network intervention with mechanism represented as both the resources deployed and the reasoning of those affected (after Dalkin 2015 [34]) and suggesting both intermediate and distal outcomes

There are, of course, many additional evaluation strategies one can use to explore the effects of our network. Gaining popularity as an approach for examining the success of complex health system change efforts is realistic evaluation [14]. In the United Kingdom, Collaborations for Leadership in Applied Health Research and Care (CLAHRCs) are efforts to create research and practice partnerships to improve services. These efforts have been the subject of research to explore the effectiveness of partners’ relationships and their ability to collaborate and ultimately change care [33]. Arguably, in contrast to the effort to deconstruct a network intervention, as we have done, the focus of realistic evaluation is to synthesise understanding and offer more unifying explanations articulated as context, mechanism and outcome statements. Commonly used to explain what has occurred context, mechanism and outcome statements can be used to propose an explanatory framework or starting theory. Here, adopting the slight modification recently suggested by Dalkin et al. [34], we offer such an explanatory proposition for the network intervention we have initiated (Fig. 2). Our proposition could be evaluated by others interested in an independent exploration of our network’s functions or effects, something we would welcome. Research on networks and their value in health and other areas has been undertaken in high-income settings [17, 33, 35–37] and may be a fruitful area of research in low- and middle-income country settings as we seek solutions to complex healthcare problems.

There are a number of limitations to the work we report. We used only a small sample of people who are all part of the CIN intervention delivery partnership to identify specific intervention strategies. We used an iterative, reflective but largely discursive approach to selecting intervention strategies and the domains of the Behaviour Change Wheel that we felt best mapped to the CIN intervention as enacted. Including a wider range of stakeholders and using a more structured consensus process, such as a Delphi approach, may have been more rigorous and could have produced somewhat different results. However, our aim was to address the question ‘What do we think we are doing’, to provide an insider’s view of an emerging theory of change. Despite these limitations, we hope the report is useful to those considering similar exercises and helps others more fully understand the CIN as a package of interventions.

## Conclusion

A network intervention was initially considered a suitable strategy for improving hospital care for children in a low-income setting based on insights from theory and an understanding of context. This intervention has been initiated and we describe here the practical form it is taking and how this can be deconstructed into a set of intervention components. These components we propose combine to help change behaviours especially those of clinical team leaders who, it is argued, are hybrid managers and should be much more engaged in improving services in general and in promoting adoption of evidence-based clinical guidelines in particular in low-income settings such as Kenya. Evaluating the effectiveness in quantitative terms of a network as an intervention is challenging. Although it spans multiple facilities, it should perhaps be considered a single entity. We intend that it does result in measurable change in delivery of recommended forms of care at a meaningful scale. Simultaneously, it should help reorient key actors in the health system to take greater responsibility for service improvement. Determining its value and the potential role of similar networks to help support adoption of recommended practices and health system change would benefit from further independent examination of the intervention approach and its successes and failures that we would welcome.

## Abbreviations

CIN: clinical information network
MUAC: mid-upper arm circumference
